# Detailed analysis of metagenome datasets obtained from biogas-producing microbial communities residing in biogas reactors does not indicate the presence of putative pathogenic microorganisms

**DOI:** 10.1186/1754-6834-6-49

**Published:** 2013-04-04

**Authors:** Felix G Eikmeyer, Antje Rademacher, Angelika Hanreich, Magdalena Hennig, Sebastian Jaenicke, Irena Maus, Daniel Wibberg, Martha Zakrzewski, Alfred Pühler, Michael Klocke, Andreas Schlüter

**Affiliations:** 1 Institute for Genome Research and Systems Biology, Center for Biotechnology, Bielefeld University, Bielefeld D-33594, Germany; 2 Department Bioengineering, Leibniz Institute for Agricultural Engineering Potsdam-Bornim, Potsdam, D-14469, Germany; 3 Computational Genomics, Center for Biotechnology, Bielefeld University, Bielefeld, D-33594, Germany

**Keywords:** Metagenome analysis, Anaerobic digester, Bacterial pathogens, Virulence determinants, High throughput sequencing, Antibiotic resistance, Biogas

## Abstract

**Background:**

In recent years biogas plants in Germany have been supposed to be involved in amplification and dissemination of pathogenic bacteria causing severe infections in humans and animals. In particular, biogas plants are discussed to contribute to the spreading of *Escherichia coli* infections in humans or chronic botulism in cattle caused by *Clostridium botulinum*. Metagenome datasets of microbial communities from an agricultural biogas plant as well as from anaerobic lab-scale digesters operating at different temperatures and conditions were analyzed for the presence of putative pathogenic bacteria and virulence determinants by various bioinformatic approaches.

**Results:**

All datasets featured a low abundance of reads that were taxonomically assigned to the genus *Escherichia* or further selected genera comprising pathogenic species. Higher numbers of reads were taxonomically assigned to the genus *Clostridium*. However, only very few sequences were predicted to originate from pathogenic clostridial species. Moreover, mapping of metagenome reads to complete genome sequences of selected pathogenic bacteria revealed that not the pathogenic species itself, but only species that are more or less related to pathogenic ones are present in the fermentation samples analyzed. Likewise, known virulence determinants could hardly be detected. Only a marginal number of reads showed similarity to sequences described in the Microbial Virulence Database MvirDB such as those encoding protein toxins, virulence proteins or antibiotic resistance determinants.

**Conclusions:**

Findings of this first study of metagenomic sequence reads of biogas producing microbial communities suggest that the risk of dissemination of pathogenic bacteria by application of digestates from biogas fermentations as fertilizers is low, because obtained results do not indicate the presence of putative pathogenic microorganisms in the samples analyzed.

## Background

Human pathogenic bacteria causing foodborne or zoonotic diseases are a major healthcare concern even in developed countries [1,2]. Usage of manure as fertilizer has been discussed as a potential source of infection. Moreover, digestates from anaerobic digesters used as fertilizers were also suspected to transfer human pathogenic bacteria onto vegetables or other crops. The recent outbreak of an enterohemorrhagic *Escherichia coli* O104:H4 strain in Germany in May 2011 is an example for a foodborne disease having vegetables as source of infection. This outbreak led to the infection of about 3,800 patients suffering from acute gastroenteritis or even the hemolytic-uremic syndrome. Epidemiological and surveillance studies were conducted at the same time by German federal institutions to identify the origin of infection. These studies led to the hypothesis that contaminated vegetables like cucumbers or tomatoes might be involved in spreading of the human pathogenic bacterium [3-5]. Press coverage also hypothesized that digestates from agricultural biogas reactors could have been a source causing these infections. Finally, fenugreek sprouts grown from seeds from Egypt were identified as the most likely source of infection [4].

However, *E. coli* is not the only relevant potential foodborne pathogen. Examples for other human pathogenic bacteria causing foodborne infections are *Listeria monocytogenes*, *Yersinia enterocolitica* or *Salmonella* species. Moreover, *Campylobacter, Vibrio* and *Clostridium* species are also known human pathogens causing foodborne diseases [1,6]. Particularly the genus *Clostridium*, which is well known to accomplish the first steps of anaerobic digestion, is widespread in biogas systems. This genus comprises some important pathogens, such as *C*. *botulinum*, *C*. *difficile*, *C*. *perfringens* and *C*. *tetani*. For instance, *C*. *botulinum* was recently identified in animal feces [7,8], a potential substrate for agricultural biogas plants. Hence, agricultural biogas plants are also accused to be involved in the spreading of *C. botulinum*[9] causing chronic botulism [10,11].

Human pathogenic bacteria are defined as bacteria causing disease in humans [12] while the term ‘virulence’ describes their degree of pathogenicity. It has been proposed that human pathogenic bacteria can enhance their virulence by acquisition of genes encoding virulence factors [12-14]. These factors may facilitate adhesion to and invasion of (specific) host cells. Moreover virulence factors can promote survival of the pathogen in the host tissue by inhibiting the immune response and increase the pathogenicity by encoding toxins. Resistance against antibiotics can also be seen as a virulence factor as it complicates medical treatment of a human pathogenic bacterial infection [14,15]. As an example, for the *E. coli* O104:H4 strain causing the outbreak in Germany it is supposed that it evolved from an enteroaggregative ancestor by acquisition of the shiga toxin encoding Stx-phage and a plasmid encoding aggregative adherent fimbriae and further virulence features [3,4].

A major substrate component used for biogas production besides agricultural plant material is manure from animals such as pigs, cattle or chicken. It is known that manure can contain potential human pathogenic bacteria such as *Salmonella* sp., *Listeria* sp., *Campylobacter* sp. or *E. coli*. Thus, spreading of manure might contribute to (zoonotic) bacterial infections [1,6,16-18]. However, several studies on lab-scale and agricultural anaerobic digesters showed that a reduction of the overall pathogen load is possible even at low temperatures [16-18]. Reduction of pathogens was shown to be very efficient for bacteria belonging to the family of *Enterobacteriaceae*, while it was less efficient for *Listeria*, *Clostridia* and *Enterococci*[16-18].

Several metagenomes of experimental and agricultural anaerobic digesters have been published recently [19-23]. These data provided insights into the microbial community involved in anaerobic digestion and methane production and into the underlying metabolic pathways.

To evaluate the risk associated with utilization of digestates from biogas plants as fertilizer on fields, the existing metagenome sequence data from different biogas reactor communities were for the first time analyzed for the presence of sequence tags originating from putative pathogenic bacteria and those representing virulence or resistance determinants.

## Results

### Searching for putative pathogens in taxonomic profiles deduced from metagenome sequence data of biogas-producing microbial communities

Origin and characteristics of metagenome sequence datasets consulted for searching of sequence tags originating from putative pathogenic bacteria are described in Table 1. Metagenomic DNA was isolated from microbial communities residing in agricultural as well as lab-scale biogas reactors at different temperatures. The taxonomic profiles of biogas-producing communities residing in the analyzed biogas reactors were computed by CARMA3 [24] and analyzed for the presence of putative pathogenic bacteria.

**Table 1:** Features of samples and corresponding biogas reactor systems analyzed in this study

**Dataset**	**Experimental setup**	**Analyzed sample**	**Reactor temperature**	**Supplied substrate**	**Reference**
B55	Two-phase reactor system	Biofilm from the anaerobic filter reactor	55°C	Rye silage, straw	[19]
S55, S65, S70	Two-phase reactor system	Digestate from the hydrolysis reactor	55°C, 65°C, 70°C	Rye silage, straw	[19]
G5, G30	Batch reactor system	Day 5 and day 30 of fermentation	37°C	Straw, hay	[20]
U1	Agricultural biogas plant, CSTR^a^	Fermentation sample	41°C	Maize silage, green rye, chicken manure	[21]

^a ^continuously stirred tank reactor.

In total, CARMA3 classified 2,183,722 environmental gene tags (EGTs), comprising all datasets, while 176,780 of these EGTs were assigned to genus and 16,035 EGTs to 351 species level. Subsequently, the profiles were examined for potentially human pathogenic distinct species (Table 2). One EGT was assigned to *C. botulinum*. This species is capable to produce the botulinum neurotoxin, which is responsible for the neuroparalytic disease botulism [25]. However, searching for sequences that are similar to the identified EGT in the NCBI non-redundant nucleotide (NT) database revealed that it encodes a part of a 23S rRNA gene of a species rather related to *C. haemolyticum* or *C. ljungahlii* (98% similarity) than to *C. botulinum*. This observation is in accordance with a recent study of methanogenic bioreactors in which pathogenic *Clostridia* could not be detected [26].

**Table 2:** EGTs assigned to putative pathogenic bacterial species and corresponding genera and orders by means of CARMA3

**Dataset**	**B55**	**S55**	**S65**	**S70**	**G5**	**G30**	**U1**	**Average**	**Average [%]**
All reads	248,775	303,493	309,589	315,387	265,256	274,138	1,347,644	437,755	100.00
All classified reads	180,454	223,536	237,134	255,499	193,025	196,763	897,311	311,960	72.26
*Clostridiales *(order)	21,479	53,756	62,570	43,940	33,353	26,989	23,482	37,939	8.67
*Clostridium*	1,535	6,622	16,459	6,326	2,855	2,163	3,333	5,613	1.28
*C. botulinum*	0	0	0	0	0	0	1	0	0.00
*C. sordelii*	0	0	0	0	0	0	0	0	0.00
*C. butyricum*	5	2	0	0	0	0	0	1	0.00
*C. difficile*	0	1	0	0	0	3	1	1	0.00
*C. perfringens*	0	0	0	0	0	0	2	0	0.00
*C. tetani*	0	0	1	0	0	0	0	0	0.00
*C. clostridioforme*	0	0	0	0	1	1	2	1	0.00
*Enterobacteriales *(order)	26	25	24	12	57	41	39	32	0.01
*Escherichia*	1	0	0	0	1	3	3	1	0.00
*E. coli*	0	0	0	0	0	0	5	0	0.00
*Salmonella*	1	0	0	0	0	2	1	0	0.00
*S. enterica*	0	0	0	0	0	0	0	0	0.00
*Shigella*	0	0	0	0	0	0	3	0	0.00
*S. boydii*	0	0	0	0	0	0	0	0	0.00
*S. dysenteriae*	0	0	0	0	0	0	0	0	0.00
*S. flexneri*	0	0	0	0	0	0	0	0	0.00
*S. sonnei*	0	0	0	0	0	0	0	0	0.00
*Lactobacillales *(order)	227	344	385	318	744	654	683	479	0.11
*Streptococcus*	19	30	30	12	149	100	193	76	0.02
*S. agalactiae*	0	0	0	0	1	1	1	0	0.00
*S. pyogenes*	0	0	0	0	0	0	0	0	0.00
*S. mitis*	0	2	0	0	2	0	0	1	0.00
*S. pneumoniae*	0	0	0	0	3	0	0	0	0.00
*S. infantarius*	0	0	0	0	2	1	5	1	0.00
*Vibrionales *(order)	5	11	12	3	11	14	11	10	0.00
*Vibrio*	1	3	2	0	2	4	15	3	0.00
*V. cholerae*	0	0	0	0	0	0	0	0	0.00
*V. fischeri*	0	0	0	0	0	0	0	0	0.00

Numbers of assignments to selected genera and orders were normalized to an equal sample size.

Moreover, a manual BLAST search of the EGTs assigned to other pathogenic species of the genus *Clostridium*, except for *Clostridium clostridioforme*, indicated that the majority of these EGTs are highly similar to related species for which pathogenicity has not been described so far. Some of the EGTs assigned to *C. clostridioforme* are identical to genes encoding hypothetical proteins originating from *C. clostridioforme*. This species has been reported to be involved in human infections, including bacteremia [27], but it also participates in fermentation of carbohydrates to acetate, lactate and formate [28]. Finally, no EGTs were classified to *Clostridium sordelii* which is a causative agent of gas gangrene.

Among the order *Enterobacteriales*, the genera *Escherichia*, *Salmonella* and *Shigella* are present in the taxonomic profiles of all biogas plant samples. No taxonomic assignments on species level were obtained for EGTs classified as *Salmonella* or *Shigella*. However, 7 EGTs exhibit a high similarity to genomic fragments originating from *Escherichia coli*. These EGTs represent a cell division component, a rhamnose-proton symporter and a DNA-damage-inducible protein. No genes encoding toxins were identified for this species.

A detailed analysis of the sequences assigned to *Streptococcus* species revealed that some EGTs encode DNA recombinases, excisionase protein transposase or hypothetical proteins that are identical in other related species. However, the EGTs assigned to *Streptococcus infantarius* are identical to the corresponding genome and different from orthologous genes in related species. The identified EGTs encode for example an isoleucyl-tRNA synthetase, N-acetylglucosamine 6-phosphate deacetylase (*nagA*) and the B subunit of DNA gyrase (*gyrB*) in *S. infantarius*, which is associated with various human infections [29].

### Mapping of metagenome sequence data to selected reference genomes of relevant pathogens

Sequence reads of the metagenomic datasets were mapped onto published genomes of pathogenic bacteria to reconstruct genomic sequences of putative pathogenic and closely related bacteria within biogas communities. Only a small number of reads of each metagenomic dataset could be mapped to the selected bacteria (Table 3). On average these reads only cover 0.1% of the respective reference genome. In contrast, more than 40% of the *Methanoculleus marisnigri* JR1 genome could be covered by reads of the U1 dataset [22]. In general, genome sequences of pathogenic strains belonging to the genus *Clostridium* feature a higher coverage by metagenomic reads than the other species. This reflects the high abundance of *Clostridia* within the microbial biogas communities [22,23].

**Table 3:** Results of mappings of metagenomic reads against selected pathogenic bacteria. The number and abundance of mapped reads per dataset and the number of covered bases and coverage are shown

	**B55**		**S55**		**S65**		**S70**		**G5**		**G30**		**U1**	
	**Mapped reads**	**Covered bases**	**Mapped reads**	**Covered bases**	**Mapped reads**	**Covered bases**	**Mapped reads**	**Covered bases**	**Mapped reads**	**Covered bases**	**Mapped reads**	**Covered bases**	**Mapped reads**	**Covered bases**
***Clostridium botulinum *A ATCC 3502**	952 (0.38%)	2,674 bp (0.05%)	1,986 (0.65%)	6,509 bp (0.18%)	2,534 (0.82%)	7,249 bp (0.18%)	2,802 (0.89%)	6,647 bp (0.18%)	1,658 (0.63%)	4,874 bp (0.13%)	1,469 (0.54%)	3,937 bp (0.10%)	9,108 (0.68%)	22,801 bp (0.56%)
***Clostridium botulinum *B1 Okra**	980 (0.39%)	2,401 bp (0.05%)	2,050 (0.68%)	6,295 bp (0.15%)	2,554 (0.82%)	8,738 bp (0.21%)	2,953 (0.94%)	5,995 bp (0.14%)	1,638 (0.62%)	3,424 bp (0.07%)	1,483 (0.54%)	3,025 bp (0.07%)	9,171 (0.68%)	21,890 bp (0.53%)
***Clostridium botulinum *C Stockholm**	801 (0.32%)	3,578 bp (0.14%)	1,779 (0.59%)	4,588 bp (0.18%)	2,284 (0.74%)	4,866 bp (0.18%)	2,431 (0.77%)	3,891 bp (0.14%)	1,401 (0.53%)	4,826 bp (0.18%)	1,254 (0.46%)	5,161 bp (0.18%)	7,807 (0.58%)	14,142 bp (0.50%)
***Clostridium botulinum *D 1873**	878 (0.35%)	3,263 bp (0.13%)	1,878 (0.62%)	4,608 bp (0.21%)	2,371 (0.77%)	7,132 bp (0.29%)	2,675 (0.85%)	3,888 bp (0.17%)	1,538 (0.58%)	3,343 bp (0.13%)	1,394 (0.51%)	3,520 bp (0.17%)	8,637 (0.64%)	24,313 bp (1.00%)
***Clostridium botulinum *E1 BoNT E Beluga**	882 (0.35%)	922 bp (0.03%)	1,801 (0.59%)	3,706 bp (0.10%)	2,406 (0.78%)	7,634 bp (0.20%)	2,663 (0.84%)	2,373 bp (0.05%)	1,612 (0.61%)	4,247 bp (0.10%)	1,402 (0.51%)	3,900 bp (0.10%)	9,209 (0.68%)	27,694 bp (0.70%)
***Clostridium botulinum *F Langeland**	965 (0.39%)	1,978 bp (0.05%)	2,044 (0.67%)	5,608 bp (0.15%)	2,563 (0.83%)	7,219 bp (0.17%)	3,023 (0.96%)	5,785 bp (0.15%)	1,623 (0.61%)	2,670 bp (0.07%)	1,481 (0.54%)	3,430 bp (0.07%)	9,252 (0.69%)	23,436 bp (0.57%)
***Clostridium butyricum *E4 BoNT E BL5262**	967 (0.39%)	7,551 bp (0.17%)	1,843 (0.61%)	5,448 bp (0.11%)	2,449 (0.79%)	6,611 bp (0.15%)	2,637 (0.84%)	4,880 bp (0.11%)	1,638 (0.62%)	3,627 bp (0.08%)	1,414 (0.52%)	4,048 bp (0.08%)	9,207 (0.68%)	28,182 bp (0.58%)
***Clostridium difficile *630**	925 (0.37%)	1,767 bp (0.05%)	1,887 (0.62%)	3,760 bp (0.09%)	2,435 (0.79%)	5,813 bp (0.14%)	2,894 (0.92%)	3,264 bp (0.07%)	1,595 (0.60%)	5,668 bp (0.14%)	1,423 (0.52%)	5,054 bp (0.12%)	8,754 (0.65%)	21,056 bp (0.48%)
***Clostridium perfringens *ATCC 13124**	914 (0.37%)	2,211 bp (0.06%)	1,854 (0.61%)	4,932 bp (0.15%)	2,400 (0.78%)	7,454 bp (0.21%)	2,733 (0.87%)	4,703 bp (0.15%)	1,554 (0.59%)	4,012 bp (0.12%)	1,367 (0.50%)	2,847 bp (0.09%)	9,007 (0.67%)	21,144 bp (0.64%)
***Clostridium tetani *E88**	922 (0.37%)	2,727 bp (0.10%)	1,917 (0.63%)	4,810 bp (0.17%)	2,520 (0.81%)	7,577 bp (0.28%)	2,933 (0.93%)	5,999 bp (0.21%)	1,563 (0.59%)	5,470 bp (0.17%)	1,415 (0.52%)	4,344 bp (0.14%)	8,970 (0.67%)	26,200 bp (0.90%)
***Escherichia coli *O104:H4 GOS1**	578 (0.23%)	2,556 bp (0.05%)	1,103 (0.36%)	2,199 bp (0.04%)	1,447 (0.47%)	2,796 bp (0.06%)	1,432 (0.45%)	1,986 bp (0.04%)	1,004 (0.38%)	4,317 bp (0.08%)	925 (0.34%)	4,002 bp (0.08%)	5,233 (0.39%)	9,149 bp (0.16%)
***Escherichia coli *O104:H4 GOS2**	584 (0.23%)	2,672 bp (0.05%)	1,137 (0.37%)	2,165 bp (0.04%)	1,470 (0.47%)	2,930 bp (0.06%)	1,587 (0.50%)	2,086 bp (0.04%)	1,030 (0.39%)	4,222 bp (0.08%)	919 (0.34%)	4,116 bp (0.08%)	5,426 (0.40%)	8,895 bp (0.16%)
***Escherichia coli *O157:H7 EC4115**	677 (0.27%)	279 bp (0.01%)	1,239 (0.41%)	1,314 bp (0.02%)	1,610 (0.52%)	848 bp (0.02%)	1,854 (0.59%)	384 bp (0.01%)	1,112 (0.42%)	1,642 bp (0.04%)	996 (0.36%)	1,010 bp (0.02%)	6,023 (0.45%)	6,932 bp (0.12%)
***Escherichia coli *O55:H7 CB9615**	678 (0.27%)	394 bp (0.01%)	1,282 (0.42%)	905 bp (0.02%)	1,666 (0.54%)	1,376 bp (0.02%)	1,805 (0.57%)	1,084 bp (0.02%)	1,146 (0.43%)	1,370 bp (0.02%)	1,023 (0.37%)	1,069 bp (0.02%)	6,008 (0.45%)	7,358 bp (0.13%)
***Salmonella enterica *subsp. enterica serovar Enteritidis P125109**	709 (0.28%)	991 bp (0.02%)	1,290 (0.43%)	1,333 bp (0.02%)	1,738 (0.56%)	1,313 bp (0.02%)	1,950 (0.62%)	632 bp (0.01%)	1,206 (0.45%)	1,889 bp (0.04%)	1,042 (0.38%)	1,312 bp (0.02%)	6,314 (0.47%)	4,543 bp (0.11%)
***Salmonella enterica *subsp. enterica serovar Typhimurium D23580**	714 (0.29%)	830 bp (0.02%)	1,285 (0.42%)	1,225 bp (0.02%)	1,714 (0.55%)	1,052 bp (0.02%)	1,909 (0.61%)	733 bp (0.02%)	1,183 (0.45%)	1,594 bp (0.03%)	1,050 (0.38%)	1,381 bp (0.02%)	6,190 (0.46%)	4,867 bp (0.10%)
***Salmonella enterica *serovar Paratyphi C RKS4594**	687 (0.28%)	364 bp (0.01%)	1,236 (0.41%)	1,035 bp (0.02%)	1,619 (0.52%)	1,298 bp (0.02%)	1,874 (0.59%)	462 bp (0.01%)	1,116 (0.42%)	1,816 bp (0.04%)	1,000 (0.36%)	996 bp (0.02%)	5,852 (0.43%)	3,744 bp (0.08%)
***Salmonella enterica *serovar Typhi Ty2**	744 (0.30%)	474 bp (0.01%)	1,307 (0.43%)	848 bp (0.02%)	1,682 (0.54%)	1,046 bp (0.02%)	1,898 (0.60%)	256 bp (0.01%)	1,191 (0.45%)	1,555 bp (0.02%)	1,024 (0.37%)	1,334 bp (0.01%)	6,242 (0.46%)	3,489 bp (0.06%)
***Shigella boydii *Sb227**	681 (0.27%)	818 bp (0.02%)	1,241 (0.41%)	1,544 bp (0.03%)	1,613 (0.52%)	676 bp (0.01%)	1,820 (0.58%)	566 bp (0.01%)	1,169 (0.44%)	1,598 bp (0.03%)	1,056 (0.39%)	1,151 bp (0.02%)	6,131 (0.45%)	7,225 bp (0.15%)
**S*higella dysenteriae *Sd197**	639 (0.26%)	560 bp (0.01%)	1,226 (0.40%)	482 bp (0.01%)	1,561 (0.50%)	1,135 bp (0.02%)	1,833 (0.58%)	613 bp (0.01%)	1,102 (0.42%)	1,818 bp (0.04%)	1,019 (0.37%)	646 bp (0.01%)	6,082 (0.57%)	7,628 bp (0.18%)
***Shigella flexneri *2a 301**	647 (0.26%)	248 bp (0.01%)	1,270 (0.42%)	489 bp (0.01%)	1,654 (0.53%)	329 bp (0.01%)	1,848 (0.59%)	112 bp (0.01%)	1,145 (0.43%)	452 bp (0.01%)	1,026 (0.37%)	967 bp (0.02%)	6,181 (0.46%)	6,211 bp (0.12%)
***Shigella sonnei *Ss046**	697 (0.28%)	663 bp (0.01%)	1,303 (0.43%)	591 bp (0.01%)	1,661 (0.54%)	376 bp (0.01%)	1,884 (0.60%)	438 bp (0.01%)	1,164 (0.44%)	1,297 bp (0.02%)	1,059 (0.39%)	1,030 bp (0.02%)	6,268 (0.47%)	9,630 bp (0.20%)
***Streptococcus agalactiae *NEM316**	713 (0.29%)	1,378 bp (0.05%)	1,295 (0.43%)	1,166 bp (0.05%)	1,692 (0.55%)	4,150 bp (0.18%)	2,134 (0.68%)	3,248 bp (0.14%)	1,149 (0.43%)	3,535 bp (0.16%)	982 (0.36%)	2,585 bp (0.12%)	6,858 (0.51%)	13,756 bp (0.58%)
***Streptococcus pyogenes *MGAS5005**	703 (0.28%)	977 bp (0.05%)	1,218 (0.40%)	1,964 bp (0.11%)	1,619 (0.52%)	2,741 bp (0.15%)	1,997 (0.63%)	2,784 bp (0.15%)	1,086 (0.41%)	2,853 bp (0.16%)	966 (0.35%)	3,039 bp (0.16%)	6,649 (0.49%)	13,704 bp (0.76%)
***Vibrio cholerae *M66**	632 (0.25%)	218 bp (0.01%)	1,122 (0.37%)	675 bp (0.01%)	1,580 (0.51%)	276 bp (0.01%)	1,829 (0.58%)	688 bp (0.01%)	1,106 (0.42%)	595 bp (0.01%)	978 (0.36%)	566 bp (0.01%)	5,888 (0.44%)	2,410 bp (0.05%)
***Vibrio fischeri *ES114**	634 (0.25%)	462 bp (0.01%)	1,134 (0.37%)	0 bp (0%)	1,510 (0.49%)	583 bp (0.01%)	1,782 (0.57%)	512 bp (0.01%)	1,084 (0.41%)	103 bp (0.01%)	921 (0.34%)	1,110 bp (0.02%)	5,925 (0.44%)	533 bp (0.01%)

Contigs and corresponding consensus sequences were extracted from the mapping datasets. Subsequently, BLAST-analyses of these sequences against organism-specific databases were performed. Assembled contigs on average are 90% identical to corresponding reference genome sequences, indicating that these biogas-producing communities analyzed only comprise strains that are related to the selected pathogenic bacteria but not identical. Moreover, functional descriptions of corresponding BLAST hits confirm these results since no pathogenicity determinants of the selected pathogenic bacteria could be detected. Most of the BLAST hits correspond to common housekeeping genes. Clostridial species within the biogas communities analyzed mostly are unknown and do not represent well-characterized species covered by database entries. In summary, sequence reads identical or almost identical to genomic sequences of selected pathogenic reference species are not present within the metagenome datasets analyzed in this study. Likewise, virulence determinants of these reference strains could not be detected.

### Searching for putative pathogenicity determinants in functional profiles deduced from metagenome sequence data of biogas-producing microbial communities by exploiting Protein Family Database (pfam) assignments

Metagenome sequence reads matching Pfam family entries representing toxins, non-toxic components of toxins and virulence determinants were analyzed. Altogether only a marginal number (0.02 – 0.04%) of the 3,064,324 metagenome sequence reads could be assigned to relevant selected Pfam families (see Table 4).

**Table 4:** Numbers and assignments of metagenomic sequences matching to toxin-associated Pfam families

**Pfam accession**	**Pfam name**	**Pathogen**	**B55**	**S55**^**a**^	**S65**^**a**^	**S70**^**a**^	**G5**^**a**^	**G30**^**a**^	**U1**^**a**^
PF05588	*C. botulinum *HA-17 protein	*C. botulinum*	27	40	35	74	25	30	32
PF05105	Holin family	*C. difficile *and others	16	17	14	27	24	16	14
PF03496	ADP-ribosyltransferase exoenzyme	*C. perfringens *and others	0	0	1	6	0	0	0

^a ^Numbers of reads are normalized to an equal sample size (sample B55).

The protein families PF05588 (*C. botulinum* HA-17 protein) as well as PF05105 (Holin family) were identified within all biogas samples (Table 4). PF05588 consists of hemagglutinin (HA) subcomponents, which are part of the L toxin, a progenitor toxin of *C. botulinum* type D strain 4947 [30]. The Pfam Holin family (PF05105) comprises TcdE/UtxA, which is involved in toxin secretion in *C. difficile*[31], but also other proteins, which are involved in bacterial lysis and virus dissemination. Interestingly, both protein families were clearly increased (PF05588, 74 EGTs, PF05105, 27 EGTs) within the hyperthermophilic digestate sample derived from the two-phase biogas system at 70°C (S70, Table 4) indicating that sanitation effect commonly assumed as consequence of increased temperatures was ineffective at least as far as clostridial species in general are concerned. Moreover, the protein family PF03496 (ADP-ribosyltransferase exoenzyme), including the ADP-ribosylating function of actin leading to lethal and dermonecrotic reactions in mammals [32], was particularly identified within the hyperthermophilic biogas samples (S70, Table 4). All other samples derived from mesophilic (38°C, 41°C) or thermophilic (55°C, 65°C) biogas reactors or batch fermentations showed a reduced number of EGTs for PF05588 and PF05105 and hardly any assignment to PF03496 (Table 4).

Beside these clostridial toxin-associated protein families, toxins derived from other bacteria (see Table 5) were not identified. For instance, the heat-labile enterotoxins (PF01375, PF01376) as well as the heat-stable enterotoxins (PF02048, PF08090) of *E. coli* were not detected within these biogas samples.

**Table 5:** Selected protein families (Pfam) used for the identification of corresponding metagenomic sequences

**Pfam accession**	**Pfam name**
PF00161	Ribosome inactivating protein
PF01123	Staphylococcal/Streptococcal toxin
PF01375	Heat-labile enterotoxin alpha chain
PF01376	Heat-labile enterotoxin beta chain
PF01742	Clostridial neurotoxin zinc protease
PF02048	Heat-stable enterotoxin
PF02258	Shiga-like toxin beta subunit family
PF02876	Staphylococcal/Streptococcal toxin
PF03278	IpaB/EvcA family
PF03318	Clostridium epsilon toxin ETX/Bacillus mosquitocidal toxin MTX2
PF03496	ADP-ribosyltransferase exoenzyme
PF03495	Clostridial binary toxin B/anthrax toxin PA
PF03505	Clostridium enterotoxins
PF05105	Holin family
PF05588	Clostridium botulinum HA-17 protein
PF05833	Fibronectin-binding protein A N-terminus
PF05946	Toxin-coregulated pilus subunit TcpA
PF06340	Vibrio cholerae toxin co-regulated pilus biosynthesis protein F
PF06511	Invasion plasmid antigen
PF07212	Hyaluronidase protein
PF07373	CAMP factor
PF07906	ShET2 enterotoxin, N-terminal region
PF07951	Clostridium neurotoxin, C-terminal receptor binding
PF07952	Clostridium neurotoxin, Translocation domain
PF07953	Clostridium neurotoxin, N-terminal receptor binding
PF07968	Leukocidin/Hemolysin toxin family
PF08090	Heat stable E. coli enterotoxin 1
PF08470	Nontoxic nonhaemagglutinin C-terminal
PF09052	Salmonella invasion protein A
PF09599	Salmonella-Shigella invasin protein C
PF10671	Toxin co-regulated pilus biosynthesis protein Q
PF12918	TcdB toxin N-terminal helical domain
PF12919	TcdA/TcdB catalytic glycosyltransferase domain
PF12920	TcdA/TcdB pore forming domain

### Searching for putative virulence determinants in metagenome sequence data implementing BLAST searches *vs.* the Microbial virulence database MvirDB

To identify possible virulence determinants within metagenome datasets of biogas-producing communities, BLAST analyses *vs*. the Microbial virulence Database MvirDB were accomplished. Metagenomic reads of each dataset were annotated based on BLASTn analyses against nucleotide sequences of the MvirDB database to identify putative virulence and resistance determinants. In total about 3.7% of all reads generated hits against sequences within the MvirDB, while about 2% of these reads featured hits against reference sequences classified as ‘virulence factor’ (Table 6). Most matching metagenomic reads were annotated as ‘virulence proteins’. Further but fewer hits corresponded to the categories ‘antibiotic resistance’, ‘transcription factor’, ‘protein toxin’ and ‘differential gene regulation’ with about 0.03 to 0.18% of all reads (Table 6). Reads annotated as ‘antibiotic resistance’, ‘protein toxin’ or ’virulence protein’ were further classified regarding their predicted function.

**Table 6:** Numbers and assignments of BLASTn analyses of metagenomic reads against nucleotide sequences of the MvirDB database

	**B55**	**S55 **^**a**^	**S65 **^**a**^	**S70 **^**a**^	**G5 **^**a**^	**G30 **^**a**^	**U1 **^**a**^
**Reads assigned**	7,054	8,736	10,247	11,805	9,481	9,457	7,597
**Status “virulence factor”**^**b**^	3,791	4,552	5,531	6,187	5,174	5,130	3,817
**Virulence protein**	3,143	3,782	4,559	5,100	4,305	4,242	3,216
**Antibiotic resistance**	332	420	510	630	479	464	328
**Transcription factor**	188	144	181	140	215	232	133
**Protein toxin**	74	104	175	222	89	100	73
**Differential gene regulation**	54	85	106	95	86	92	66

^a ^Numbers of reads are normalized to an equal sample size (sample B55).

^b ^As defined in the MvirDB database.

#### Protein toxins

Among the total number of metagenome sequence reads obtained for the different biogas reactors, only about 0.02 to 0.08% represent genes encoding different protein toxins (Table 7). A total of 67 different protein toxins were identified within the datasets by sequence similarity. Most of the detected protein toxins were assigned to the group of exotoxins and within this subgroup subtilisins, hyaluronidases, hemolysins and RTX toxins were annotated.

**Table 7:** Numbers and assignments for reads annotated as “protein toxin” based on MvirDB classifications

		**B55**	**S55**^**a**^	**S65**^**a**^	**S70**^**a**^	**G5**^**a**^	**G30**^**a**^	**U1**^**a**^
**Exotoxins**	Subtilisin	35	48	100	118	42	43	39
	RTX	14	30	38	63	13	14	20
	Hyaluronidase	5	1	1	0	0	0	1
	Hemolysin	2	5	12	17	10	6	4
	Others	1	1	1	1	0	2	2
**Endotoxins**	LPS	7	11	4	0	7	19	2
	Others	2	0	2	0	2	2	0
**Others**		8	8	18	25	15	15	6
**Total**		74	104	175	222	89	100	73

^a ^Numbers of reads are normalized to an equal sample size (sample B55).

Within these exotoxins, 37 different subtilisins and subtlilisin-like serine proteases were detected by sequence similarity and accordingly constitute the most prominent subgroup within the detected protein toxins. Corresponding proteases are present in microorganisms and even in higher eukaryotes [33]. Some subtilisins function as scavengers for nutrients [34,35] or their proteolytic properties are activated during pathogenesis in plants [36]. Risk assessment by the Toxic Substances Control Act of *B. subtilis*, one of the main producers of subtilisin, revealed that the protease only shows very low toxigenic properties. However, subtilisin is able to cause allergic reactions. The fact, that subtilisins are commonly used in different detergents may be interpreted in a way that subtilisin production by biogas community members does not pose an imponderable hazard to the environment or human health.

The second subgroup of exotoxins detected in every biogas sample comprises RTX toxins. The number of reads assigned to corresponding protein toxins varies between 13 and 63 representing only three different RTX genes. RTX toxins contribute to pathogenicity by interacting with the host’s immune system [37]. The gene products of the three different RTX genes detected are involved in the transport of the corresponding exotoxins, which were not verifiably within any sample.

In four of the biogas reactors, hyaluronidase genes probably originating from the species *C. perfringens* were detected. This species is a ubiquitous environmental organism [38] and a common human and livestock pathogen, causing gastroenteritis and gas gangrene in humans [39]. The number of detected sequences assigned to this gene family is relatively low and only ranges between 1 and 5 hits.

Altogether four different hemolysin genes were traceable in a low amount within each sample. Hemolysins are cytotoxic proteins that destroy the integrity of the host cell membrane by different mechanisms. The function of these hemolysin toxins is aimed at nutrient acquisition mostly by lysing leukocytes of the host [40]. Among the hemolysin genes identified in the datasets analyzed, the gene *hlyC* is present as deduced from sequence similarity analyses. The *hlyC* gene product activates the pore forming hemolysin HlyA in an unknown way [41]. However, *hlyA*-like genes were not detectable in the metagenome data. Additionally remaining possible and pore-forming hemolysins were not identified within the present data.

Only one to two reads per metagenome dataset were assigned to other exotoxin genes. Moreover, four different genes predicted to be involved in lipopolysaccharide (LPS) synthesis from the human stomach pathogen *Helicobacter pylori* were detected within six datasets. LPS originating from this pathogen mimics human glycan structures and contributes to the virulence by modulation of the immune system [42].

Overall only a low number of reads feature similarity to sequences categorized as ‘protein toxin’. Moreover, reference proteins encoded by these sequences are known to possess a low degree of toxicity.

#### Virulence proteins

The assignments of MvirDB entries classified as ‘virulence protein’ show a great diversity regarding their function. However, some of these annotations were present at high abundance in all datasets (see Table 8). Among these some may play a role in stress response (endopeptidase Clp ATP-binding chain C, ATP-dependent Clp protease ATP-binding subunit ClpX, ClpB protein, DNA mismatch repair protein, chaperonin GroEL) [43,44], sugar and energy metabolism (pyruvate kinase, GTP pyrophosphokinase, UDP-N-acetylglucosamine 2-epimerase) or are thought to have further functions not directly related to virulence (carbamoyl-phosphate synthase large chain, putative lysil-tRNA synthetase LysU). At first view, corresponding genes mediate general features of microorganisms and do not pose a potential risk regarding virulence. However, some of these genes are described to be involved in virulence of certain bacteria. For example the Clp ATPase and proteases are involved in quality control of proteins and their structure [44] in non-stress as well as in stress situations and are needed for cellular differentiation. Hence, these enzymes most probably also ensure the survival of cells in pathogenic interactions [44]. Moreover, they regulate the expression of further virulence determinants.

**Table 8:** Numbers and assignments for reads annotated as “virulence protein” based on MvirDB classifications

	**B55**	**S55**^**a**^	**G5**^**a**^	**G30**^**a**^	**S65**^**a**^	**S70**^**a**^	**U1**^**a**^
Endopeptidase Clp ATP-binding chain C	100	139	162	149	162	227	124
Carbamoyl-phosphate synthase large chain	77	95	73	68	167	156	53
Chaperonin GroEL	65	102	113	91	115	139	109
Putative lysil-tRNA synthetase LysU	63	62	86	78	85	92	76
DNA mismatch repair protein	52	59	93	92	72	112	69
GTP pyrophosphokinase	49	60	41	48	75	95	49
ATP-dependent Clp protease ATP-binding subunit ClpX	46	82	78	80	102	99	61
UDP-N-acetylglucosamine 2-epimerase	43	0	0	0	0	0	24
ClpB protein	41	57	79	65	50	98	62
Pyruvate kinase	37	40	37	0	49	0	38

^a ^Numbers of reads are normalized to an equal sample size (sample B55).

Accordingly, presence of metagenomic reads sharing similarity to those genes described to be involved in bacterial virulence does not allow drawing the conclusion that virulent bacteria reside in microbial communities of the samples analyzed because a read based analysis *per se* cannot take into account the genomic context of a bacterium harboring a putative virulence determinant. Certainly, a putative virulence gene in a pathogenic organism might be more severe than the same gene in an otherwise harmless bacterium.

#### Antibiotic resistance determinants

**Figure 1:**
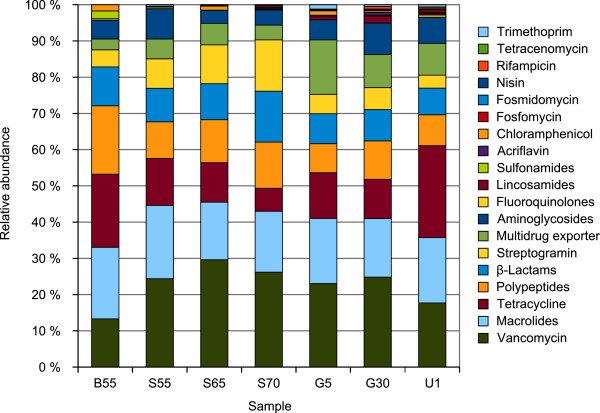
**Relative abundances of reads annotated to have a predicted function in the context of resistance to antimicrobial drugs. **Annotations by means of BLASTn analyses of metagenomic reads against the MvirDB identified about 0.09% (B55) to 0.22% (S70) of metagenome sequence reads to confer resistances against groups of or specific antibiotics or to encode putative multidrug exporters.

About 0.09% (B55) to 0.22% (S70) of metagenome sequence reads were annotated to have a predicted function in the context of resistance to antimicrobial drugs. Corresponding annotations mainly represent eight groups of antimicrobial compounds for which resistance determinants were identified (Figure 1). These groups comprise vancomycin, macrolide, tetracycline, polypeptide (bacitracin, polymyxin), β-lactam, streptogramin and aminoglycoside (kasugamycin, streptomycin, kanamycin, spectinomycin) resistance determinants as well as multidrug exporter components. Further refer to resistances against a number of additional antibiotics (Figure 1). No clear differences concerning the abundance of specific resistance types can be observed between the samples (Figure 1). Moreover, annotated resistances are based on different mechanisms [45] including enzymatic inactivation of the drug (beta-lactames, amidoglycosides), mutational alteration of the target protein (fluoroquinolones), acquisition of genes encoding gene products that are less susceptible to the antibiotic (trimethoprim), bypassing the target of antimicrobial action (vancomycin) or by prevention of drug access to the target (multidrug efflux pumps). Especially for the last four resistance mechanisms the approach to predict the existence of resistance determinants by means of similarity searches in curated databases such as MvirDB has limitations because reliable functional conclusions cannot be drawn. For example, reads annotated as multidrug exporters might encode pumps for the transport of compounds that do not act as antibiotics or reads annotated as products less susceptible to a drug might encode a drug sensitive target. Surprisingly, a high number of reads were annotated to have a predicted function in vancomycin resistance. Vancomycin binds to the D-Ala-D-Ala termini of peptidoglycan intermediates and inhibits the crosslinking of the peptidoglycan layer [46]. Some bacteria (such as *Enterococci* or *Leuconostoc mesenteroides*) are resistant to vancomycin because their cell wall does not contain the D-Ala-D-Ala but D-Ala-D-Lactate termini instead. Enzymes involved in the formation of each type of termini are closely related ligases [46,47] which may again lead to the annotation of reads encoding D-Ala-D-Ala ligases as vancomycin resistance determinants. These intrinsic limitations might cause an overestimation of reads involved in antibiotic resistance.

Overall a variety of putative antibiotic resistance determinants was identified. However, their abundance within each metagenome dataset is quite low.

## Discussion

Biogas plants are discussed to contribute to the proliferation and dissemination of pathogenic bacteria and pathogenicity/virulence determinants in the environment since digestates from biogas reactors are applied as fertilizer on fields. This practice bears the risk that pathogens residing in digestates contaminate crops and vegetables that serve as food for animals and humans thus abetting zoonotic diseases. To our knowledge, in this study metagenome sequence data were analyzed for the presence of sequence tags indicative for the occurrence of pathogens or pathogenicity/virulence determinants for the first time. The sensitivity and resolution of this kind of approach should be very high since it is based on nucleotide sequence data. Moreover, this approach is less biased compared to methods based on PCR for detection of pathogenicity determinants or cultivation of putative pathogens.

Inspection of taxonomic profiles deduced from metagenome sequence data and mapping results on pathogenic reference genomes does not elucidate strong evidence for the presence of pathogens within fermentation samples of biogas reactors. Sequence tags originating from pathogenic members of the family *Enterobacteriaceae* could hardly be detected within the metagenome data analyzed which is in accordance with earlier studies based on microbiological and molecular genetic methods applied for detection of species belonging to this group of pathogens [16-18]. Hence, survival of enterobacterial species seems to be drastically reduced in biogas fermentations. Sanitation under thermophilic conditions might occur. However, this effect is not visible from our data, since even mesophilic conditions in fermentation seem to be non-permissive for *Enterobacteria*. Likewise, clostridial pathogens are absent in the samples analyzed in this study which also is line with previous results obtained for experimental methanogenic bioreactors [26]. The authors of the latter study concluded that neither pathogenic *Clostridium* species nor *Clostridia* closely related to pathogenic ones could be detected in their samples [26]. Occurrence of pathogens such as *Clostridium clostridioforme* and *Streptococcus infantarius* in biogas fermentation samples should specifically be addressed in future studies since few identical EGTs were identified in the metagenome datasets analyzed here. *C. clostridioforme* appeared to be associated with serious or invasive human infections including bacteremia [27] whereas *S. infantarius* can be isolated from traditionally fermented dairy and plant products and holds a potential health risk for animals and humans [29]. Regarding the latter species, a residual risk remains when applying digestates as fertilizer. It should also be noted here that the metagenomes of this study were not sequenced to saturation. Accordingly, rare pathogens might not have been detected due to low coverage of their genomes within the metagenome sequence datasets. Moreover, it has to be considered that some of the reactors sampled in this study were not continuously fed with manure. Hence, the pathogenic load in reactors regularly fed with manure – especially pig manure – could be higher. In future studies regarding detection of pathogens in biogas fermentation samples, gene-centered approaches applying high-throughput sequencing would be appropriate to identify specific rare pathogens. In this context, 16S rRNA gene amplicon sequencing or PCR-based analysis of pathogen-specific signature genes should be considered. It should also be taken into account that genomic traces identified in this study might not be in an active state anymore. Hence, *in situ* analyses along with metagenome analysis might further improve detection of pathogens in this context.

Concerning identification of sequence tags representing bacterial toxins and virulence determinants, it has to be taken into account that the genomic context of organisms encoding these determinants is of importance. For example, virulence determinants that are present in a non-pathogenic species most probably are harmless, whereas these genes may enhance virulence of pathogens. Metagenome studies intrinsically do not allow drawing any reliable conclusions regarding the genomic context of particular determinants. Accordingly, possible detection of toxin genes and virulence determinants only allows for very vague assessments concerning the presence of pathogens. However, antibiotic resistance determinants may be released with digestates and hence spread in the environment. Since in most biogas reactors manure from cattle or pigs is used as substrate, antibiotic resistant bacteria selected by application of antimicrobial treatments will end up in biogas plants where resistance determinants located on mobile genetic elements potentially can be transferred to biogas community members and finally be released with digestates into the environment. It cannot be excluded that bacteria harboring resistance determinants occasionally get incorporated by humans. However, prediction of resistance determinants by sequence similarity based methods clearly leads to an overestimation of resistance determinants since in principle functionality of these determinants remains unclear. It should also be noted here that direct application of manure from cattle or pigs as fertilizer on fields is a commonly accepted agricultural practice.

In summary, detection of putative pathogenic bacteria exploiting metagenome sequence data currently is the most reliable approach addressing this issue. However, the informative value of the method clearly depends on careful selection of pathogen-indicative determinants. Results of this study revealed that the risk of unintended proliferation of pathogens in biogas fermentations and their dissemination in the environment is rather low.

## Methods

### Datasets

Seven metagenomic datasets from different experimental and agricultural biogas reactors were analyzed for the presence of putative pathogenic bacteria (Table 1). The samples B55, S55, S65 and S70 were taken from an experimental two-phase leach-bed biogas reactor. This system consisted of a leach-bed reactor, a leachate reservoir and an anaerobic filter reactor, which was described recently [19]. The reactor was inoculated with manure after it was brought into service. Since then it has been fed with rye silage and straw. The samples S55, S65 and S70 derived from the digestate of the leach-bed reactor at 55°C, 65°C and 70°C, respectively, whereas B55 was taken from a packing of the anaerobic filter reactor at 55°C. The samples G5 and G30 derived from a 30-day anaerobic digestion batch test (37°C) with recalcitrant substrate taken at day 5 and day 30. Here, digestates of an anaerobic digester supplied with maize and manure was mixed with low amounts of straw and hay. Finally, the sample U1 derived from a mesophilic (41°C) agricultural biogas plant supplied with maize silage, green rye and low amounts of chicken manure [21].

The libraries, which were created from the isolated metagenomic DNAs, were sequenced on the Genome Sequencer (GS) FLX platform applying the FLX Titanium sequencing chemistry (Roche Applied Science). Raw data were processed by means of the analysis pipeline for whole genome shotgun sequence reads applying the GS FLX System Software (version 2.6).

### Taxonomic profiles

The metagenome sequences obtained from the different samples were classified using the BLASTx-approach of CARMA3 [24] in order to determine the prevalence of potentially human pathogenic bacteria. For this purpose, CARMA3 was executed using standard settings. Afterwards, the profile was evaluated for the presence of selected species that are associated with infections in humans (species of the genera *Escherichia*, *Streptococcus*, *Vibrio*, *Clostridium*, *Salmonella* and *Shigella*). Finally, identified environmental gene tags (EGTs) were manually searched for homologue matches in the NCBI non-redundant nucleotide (NT) database using standard BLAST settings [48].

### Genome mappings

The metagenomic reads of the different datasets were aligned to chromosomal sequences of selected pathogenic bacteria (Table 9) by means of the gsMapper program (Roche Genome Analyzer Data Analysis Software Package, version 2.6) in order to confirm the presence of their virulence determinants. Default settings of the gsMapper (90% sequence identity, 40 bp overlap) were used to also map reads originating from closely related species. Multiple contigs and corresponding consensus sequences were generated from the mapped reads. To identify virulence determinants of selected reference strains in the metagenomic datasets, a BLAST-analysis of the resulting contigs was performed. The BLAST-analysis was done with rather relaxed settings (E-value: 1*10^-4^, sequence identity: 80%), but with organism-specific BLAST-database. The results were then analyzed for the presence of known pathogenic determinants.

**Table 9:** Selected pathogenic reference strains for genome mappings of metagenomic sequences and their features

**Species**	**Accession number**	**Genome size [Mbp]**	**Sequence status**	**Disease**
*Clostridium botulinum *A str. ATCC 3502	[GenBank:NC_009495]	3.90	Finished	Botulism
*Clostridium botulinum *B1 Okra	[GenBank:NC_010516]	4.10	Finished	Botulism
*Clostridium botulinum *C Stockholm	[GenBank:NZ_AESA00000000]	2.77	draft genome	Botulism
*Clostridium botulinum *D 1873	[GenBank:NZ_ACSJ00000000]	2.40	draft genome	Botulism
*Clostridium botulinum *E1 BoNT E Beluga	[GenBank:NZ_ACSC00000000]	4.00	draft genome	Botulism
*Clostridium botulinum *F Langeland	[GenBank:NC_009699]	4.01	Finished	Botulism
*Clostridium butyricum *E4 BoNT E BL5262	[GenBank:NZ_ACOM00000000]	4.76	draft genome	Botulism
*Clostridium difficile *630	[GenBank:NC_009089]	4.30	Finished	Diarrhea and colitis
*Clostridium perfringens *ATCC 13124	[GenBank:NC_008261]	3.26	Finished	Gas gangrene
*Clostridium tetani *E88	[GenBank:NC_004557]	2.87	Finished	Tetanus
*Escherichia coli *O104:H4 str. GOS1	[GenBank:AFWO00000000]	5.31	draft genome	Hemolytic-uremic syndrome
*Escherichia coli *O104:H4 str. GOS2	[GenBank:AFWP00000000]	5.31	draft genome	Hemolytic-uremic syndrome
*Escherichia coli *O157:H7 str. EC4115	[GenBank:NC_011353]	5,70	Finished	Hemorrhagic colitis
*Escherichia coli *O55:H7 str. CB9615	[GenBank:NC_013941]	5.45	Finished	Gastroenteritis
*Salmonella enterica *subsp. enterica serovar Enteritidis str. P125109	[GenBank:NC_011294]	4.69	Finished	Salmonellosis
*Salmonella enterica *subsp. enterica serovar Typhimurium str. D23580	[GenBank:NC_016854]	4.88	Finished	Gastroenteritis
*Salmonella enterica *serovar Paratyphi C RKS4594	[GenBank:NC_012125]	4.89	Finished	Paratyphoid fever
*Salmonella enterica *serovar Typhi Ty2	[GenBank:NC_004631]	4.79	Finished	Typhoid fever
*Shigella boydii *Sb227	[GenBank:NC_007613]	4.65	Finished	Dysentery
*Shigella dysenteriae *Sd197	[GenBank:NC_007606]	4.56	Finished	Dysentery
*Shigella flexneri *2a str. 301	[GenBank:NC_004337]	4.83	Finished	Dysentery
*Shigella sonnei *Ss046	[GenBank:NC_007384]	5.06	Finished	Dysentery
*Streptococcus agalactiae *NEM316	[GenBank:NC_004368]	2.21	Finished	Neonatal GBS meningitis
*Streptococcus pyogenes *MGAS5005	[GenBank:NC_007297]	1.84	Finished	Wide range of infections
*Vibrio cholerae *M66	[GenBank:NC_012578]	3.94	Finished	Cholera
*Vibrio fischeri *ES114	[GenBank:NC_006840] [GenBank:NC_006841]	4.27	Finished	-

### Identification of reads with similarity to toxin protein families

The Pfam database encompasses altogether 13,672 protein families also including toxic protein families and virulence determinants, which are all represented by multiple sequence alignments and hidden Markov models [49]. Different protein families relevant for toxicity of *Clostridium* sp., *E*. *coli*, *Streptococcus* sp., *Staphylococcus* sp., *Shigella* sp. and *Vibrio* sp. were identified (Table 5) using the Pfam database version 26.0 [49]. Seed sequences matching these Pfam domains were extracted from the Pfam database. The metagenomes were then screened for the presence of these factors based on a BLASTx analysis (e-value cutoff: 1*10^-20^) and annotated according to their best hit. The results were then checked for the Pfam accessions of interest. The stringent cutoff was applied because metagenomic reads typically represent gene fragments which is due to short read lengths. Therefore it is important to apply stringent cutoffs to avoid false positive assignments caused by conserved domains. The length of a query sequence often does not suffice to distinguish between hits to conserved domains (false positives) and full-length gene alignments. Thus, a more stringent cutoff is required when analysing short reads compared to analyses involving full-length genes. Additionally, the sequence database applied is a comparatively small database which also requires a more stringent cutoff to exclude false positive hits.

### BLASTn vs. the microbial virulence database MvirDB

Metagenomic sequences were screened for the presence of gene fragments encoding putative virulence factors based on a BLAST search versus the MvirDB database [14] using an e-value cutoff of 1*10^-20^ and annotated with the best hit to analyze the presence of further and previously not selected putative virulence determinants. Only those hits against database entries categorized as “virulence factor” were used for further analysis. Hits against database entries classified as “protein toxin”, “antibiotic resistance” or “virulence protein” were further classified regarding their function.

## Abbreviations

MvirDB: Microbial Virulence Database; BLAST: Basic Local Alignment Search Tool; RTX: Repeats in Toxin; PCR: Polymerase Chain Reaction; Stx: Shiga toxin; EGT: environmental gene tag; NT: NCBI non-redundant nucleotide database; LPS: lipopolysaccharide; GS: Genome Sequencer; Pfam: Protein families; CSTR: Continuously stirred tank reactor.

## Competing interests

The authors declare that they have no competing interests.

## Authors’ contributions

FGE participated in the search of putative virulence determinants in the metagenome sequence data and helped to draft the manuscript. AR and AH searched for putative pathogenicity determinants in functional profiles by exploiting Pfam assignments. MH and IM were involved in the search of putative virulence and resistance determinants in the metagenome sequence data. SJ and MZ managed metagenome sequence data, initiated computational analyses and performed bioinformatic analyses by means of BLAST, MetaSAMS and CARMA3. MZ deduced taxonomic profiles from metagenome sequence data and searched for putative pathogens. DW mapped metagenome sequence data on selected reference genomes. AP, MK and AS conceived of the study, coordinated analyses, and formed the paper concept. All authors contributed to writing of the manuscript, read and approved the final manuscript.
